# Anti-IL1 therapy in patients with refractory FMF living inGermany

**DOI:** 10.1186/1546-0096-13-S1-P110

**Published:** 2015-09-28

**Authors:** B Buhl, H-M Lorenz, N Blank

**Affiliations:** 1 University Hospital Heidelberg, Internal Medicine 5, Division of Rheumatology, Heidelberg, Germany

## Introduction

About 10-20% of patients with familial Mediterranean fever (FMF) show an inadequate response to colchicine. Patients with colchicine-resistant FMF with or without AA-Amyloidosis can be treated with Interleukin-1 (IL-1)-inhibiting drugs.

## Objective

We report our experience in adult patients with colchicine-resistant FMF who were treated with anakinra or canakinumab.

## Patients and methods

Demographic data, clinical and laboratory parameters, MEFV mutations, patient reported outcomes and physician global health were analyzed in 15 patients treated with anakinra or canakinumab.

## Results

Within our cohort of 160 adult patients with FMF, we identified 15 patients (4 female and 11 male) who were treated with anakinra (n=13) or canakinumab (n=2). Twelve of 15 patients (80%) were of turkish-armenian ancestry. The median FMF severity score was 8 (range 5-14). Patients carrying two high-penetrance MEFV mutations (M694V or M680I) had a severity score of 9 (8/15=53%). Patients with a single high penetrance mutation had a severity score of 11 (3/15=20%). Four patients (4/15=27%) had no MEFV mutations and the FMF severity score was 7.5 (p=0.2). FMF-related AA amyloidosis was diagnosed in 6 patients (40%) and the median FMF severity score was 10 compared to a severity score of 7 in 9 patients without amyloidosis (60%) (p=0.3). Anakinra was used continuously in 13 patients and in 2 patients only during attacks. The number of FMF attacks was significantly reduced by anti-IL1 treatment (p=0.0024). The patient reported health and the physician reported global health were both improved significantly (p

## Conclusion

IL-1-blocking therapies are well tolerated and effective in patients with colchicine-resistant FMF. Blocking IL-1 reduced the number and severity of FMF attacks.

